# Improving Fall Classification Accuracy of Multi-Input Models Using Three-Axis Accelerometer and Heart Rate Variability Data

**DOI:** 10.3390/s25041180

**Published:** 2025-02-14

**Authors:** Seunghui Kim, Jae Eun Ko, Seungbin Baek, Daechang Kim, Sungmin Kim

**Affiliations:** 1 Department of Regulatory Science for Medical Device, Dongguk University, Seoul 04620, Republic of Korea; ksh99110@naver.com (S.K.); 2018111721@dgu.ac.kr (J.E.K.); 2 Department of Medical Device Business, Dongguk University, Seoul 04620, Republic of Korea; bjh5863@gmail.com; 3 Department of Biomedical Engineering, Dongguk University, Goyang-si 10326, Republic of Korea; kimdaechang10@gmail.com

**Keywords:** three-axis acceleration sensor, Holter electrocardiograph, multi-input model, fall classification, heart rate variability (HRV)

## Abstract

Reduced body movement and weakened musculoskeletal function as a result of aging increase the risk of falls and serious physical injuries requiring medical attention. To solve this problem, a fall prevention algorithm using an acceleration sensor has been developed, and research is being conducted to enable continuous monitoring using a Holter electrocardiograph. In this study, we implemented a multi-input model that can detect and classify movements, including falls, utilizing the baroreflex characteristics of the heart’s potential energy changes due to movement, measured with an electrocardiogram with a three-axis acceleration sensor and a Holter electrocardiograph. Patterns were identified from the various movement characteristics of acceleration sensor data using a deep learning model consisting of CNN-LSTM, and heart rate variability (HRV) data were analyzed using a wide learning model to provide additional weight values for fall classification. Finally, a multi-input model using wide and deep learning was proposed to enhance the accuracy of fall classification. The results show that the HRV increased in fall case except in two motion types, while it decreased when standing up from a chair, indicating the application of the baroreflex characteristics reflecting the heart’s potential energy. Compared to the classification model using conventional HRV and ACC, a higher accuracy was achieved in the multi-input model using ACC-HRV data, and a precision, recall, and F1 score of 0.91 was measured, indicating improved performance. This is expected to have a positive impact on fall prevention by improving the accuracy of fall classification in the elderly for 15 different movements.

## 1. Introduction

The increasing portion of the global population made up of older adults is creating significant social and economic challenges. According to Statistics Korea, the elderly population is projected to constitute 46.4% of the total population by 2070, highlighting the critical need to adapt to demographic shifts and develop proactive strategies to address the challenges of an aging society [1]. Furthermore, the global population aged 65 and older is expected to more than double, rising from approximately 770 million in 2021 to 1.6 billion by 2025, with the population of individuals aged 80 and older growing at an even faster rate. Population aging is an irreversible global trend.

Aging itself presents the greatest risk to older adults. Beyond the physical changes associated with aging, trauma resulting from environmental factors is a leading cause of safety incidents in this population [1,2]. Notably, falls represent the largest proportion of safety incidents among older adults, accounting for 62.7% of such incidents between 2016 and 2023, with approximately 7.2% of individuals aged 65 years and older experiencing a fall. The frequency of falls also tends to increase with age [3]. Among older adults presenting to emergency departments following a fall, 80.9% reported serious or severe injuries requiring medical intervention, including head, brain, leg, hip, and lower back injuries, which can be life-threatening [1]. In the United States, unintentional falls have been identified as the leading cause of injury-related death among individuals aged 65 and older [4,5,6,7].

To mitigate the risk of death, rising medical costs, economic losses, and the broader social impacts caused by falls, research on fall classification has gained global momentum. Various algorithms have been developed to monitor human activity and classify falls using acceleration sensors [4,5,6,7,8,9,10,11,12,13,14,15]. While different methods are employed in fall classification, time-domain analysis based on triaxial acceleration sensors is widely adopted for real-time fall classification in everyday life [16,17,18,19,20,21,22,23,24]. These sensors measure acceleration (ACC), a physical quantity which represents the rate of change in velocity over time. Consequently, algorithms and systems are being developed to classify falls by analyzing movement along the *x*, *y*, and *z*-axes [25,26,27,28,29,30].

However, current systems are limited by the fact that they only cover a few minimal movements such as walking, sitting, and falling, which do not reflect real-world movements [11,12], or by the fact that the number of subjects is significantly low [10,11,12,31]. In addition, existing studies using open datasets have mainly used triaxial ACC data or inertial sensors to classify falls, while a number of studies have used gyroscopes and magnetometers instead [32,33,34,35,36,37,38]. However, these studies are limited by a restricted number of subjects (24, 10, 16, 3, 17, etc.) and a relatively small range of motion for fall classification (13, 8, 6, 11, etc.) [13,14,15].

To compensate for these limitations, this study aims to improve the accuracy of a model that classifies 15 types of movements and falls by integrating ECG data and three-axis acceleration data collected from 210 subjects. To this end, we used a deep model to reflect the various movement characteristics of acceleration sensors to find patterns in artificial intelligence and a wide model to provide additional weights to the model using heart rate variability, which represents the characteristics of changes in the heart rate, to take advantage of the pressure reflection characteristics of the cardiovascular system [39,40]. In particular, as a difference from previous studies, we used HRV data to reflect physiological changes related to movement [41], enhancing the discrimination between similar movements which are difficult to distinguish using existing ACC data alone. In addition, this study used a large dataset of 13,650 data points from a total of 210 subjects, collected through Holter electrocardiographs attached to the heart to secure high data reliability compared to previous studies.

As a result, we ultimately developed a multi-input model using wide and deep learning to improve classification accuracy for 15 types of movements, particularly by effectively classifying falls after they occurred.

## 2. Materials and Methods

### 2.1. Physiological Changes and Electrocardiograms

An electrocardiogram (ECG), which can be recorded using a Holter electrocardiograph, captures the heart’s electrical activity generated by its contraction and relaxation phases [42]. The rhythmic beating of the heart exhibits periodicity, and variability in this periodicity over short intervals is referred to as HRV. HRV is influenced by physiological changes and the body’s homeostasis, often resulting from the interactions between the sympathetic and parasympathetic nervous systems [43,44,45].

### 2.2. Heart Rate Variability

**Figure 1:**
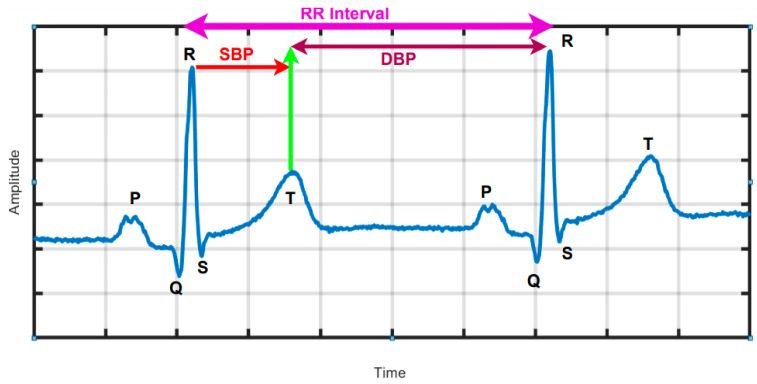
Calculating HRV using ECG data. (P: Atrial contraction, QRS complex: Ventricular contraction, T: Ventricular relaxation).

ECGs are used to monitor the heart rate and electrical activity of the heart, which allows for the calculation of the HRV. The HRV is a measure of the variability between heartbeats, which can be measured by the change in the minute temporal interval between one heart cycle and the next (RR Interval), as shown in Figure 1. It is a metric that analyses changes in the time interval between heartbeats and plays an important role in assessing the state of the autonomic nervous system and cardiovascular health [46,47].

(1) $$R-R\;intervals\;(RR)=\frac{1}{N-1}\sum_{i=1}^{n-1}\left(RR_{i}\right)$$

**Theorem** **1.** *Formula for calculating r–r intervals.*

It can also be affected by changes in posture, baroreflex responses to exercise-induced blood pressure fluctuations, and autonomic modulation via the nucleus accumbens [46,48,49]. Clinically, head-up tilt tests and preliminary studies have demonstrated that changes in HRV, reflecting cardiac potential energy variations, can be detected. Therefore, we hypothesized that HRV, in combination with triaxial acceleration sensors, could serve as valuable data for movement classification models [12,50,51].

### 2.3. Collection of Acceleration and ECG Data

**Figure 2:**
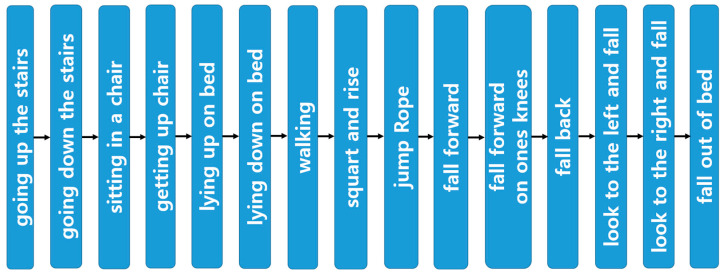
Data measurement process.

**Figure 3:**
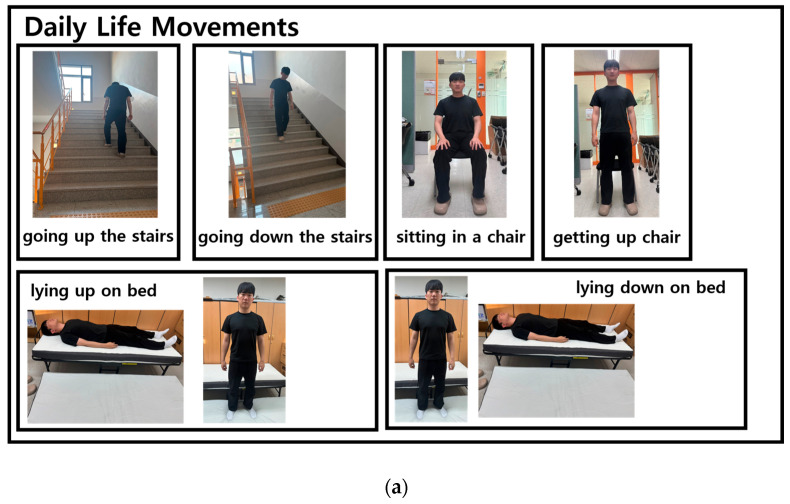

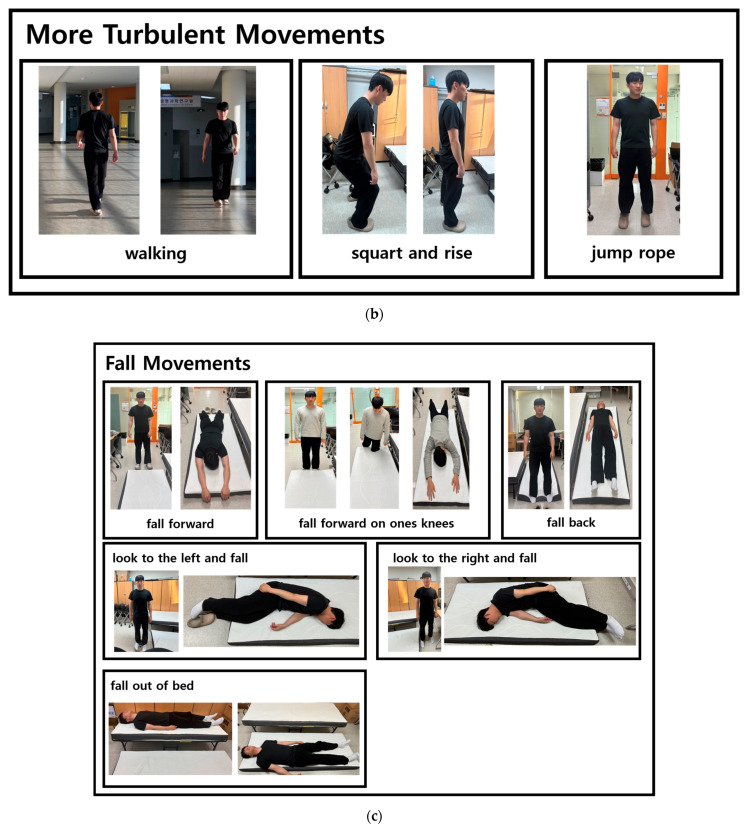
Data measurement of movements: (**a**) daily life movements; (**b**) more turbulent movements; and (**c**) fall movements.

Informed consent was obtained from all the participants in this study, which had been approved by the Severance Clinical 1-2023-0006. Final consent for the use and publication of the data were also obtained. Participants were selected based on their ability to perform activities of daily living, engage in physical exercises, and replicate falls. A total of 210 participants, comprising 115 males and 95 females, were recruited, with demographic details provided in Table 1. Fifteen distinct movements were selected for the fall classification model, including stair climbing, stair descent, sitting, standing, lying down, and lying prone activities commonly performed in daily life. Additionally, movements such as walking, squatting, and hopping in place were included to capture more turbulent motions. The fall movements consisted of falling forward, falling to one knee, falling backward, falling to the right, falling to the left, and falling out of bed. Each movement was performed five times for 10 s, as shown in Figure 2 and Figure 3, with a rest period of 1 min after each movement before repeating the following movement.

**Figure 4:**
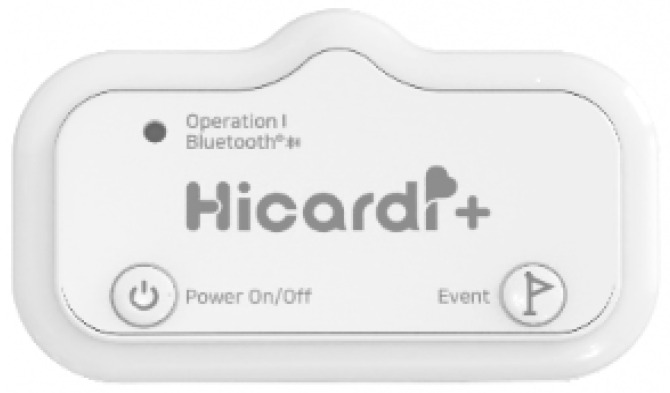
Mezoo’s Holter electrograph ‘HiCardi+’.

**Figure 5:**
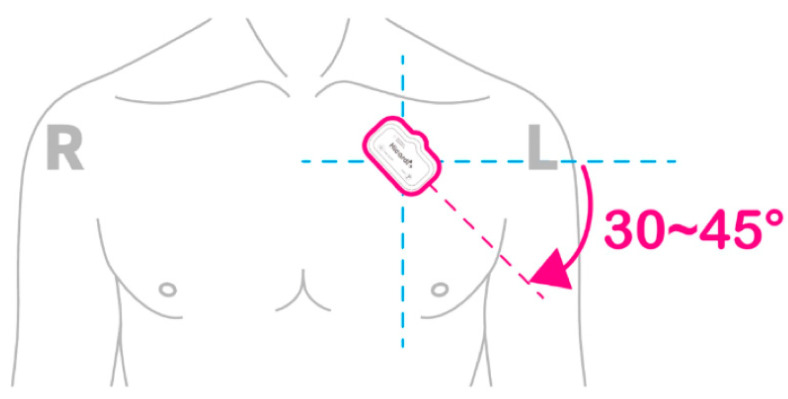
The attachment location of the HiCardi+.

To measure ECG data and acceleration sensor change data, we used the HiCardi+ patch-type Holter electrocardiograph from Mezoo’s (Wonju-si, South Korea), shown in Figure 4 (article licence number: no. 22-4085, IAR Embedded Workbench 7.40.7 Tool). It is registered as a medical device and can simultaneously measure various vital signs such as ECG, respiration, temperature, and acceleration data. After attaching the device’s specialized electrodes, it was positioned on the flat region on the left side of the chest, just below the collarbone, at the designated attachment point, as shown in Figure 5. The HiCard+ device captured data for three-axis ACC and ECG at sampling rates of 25 Hz and 250 Hz, respectively. To synchronize the data for analysis, a resampling method was applied to the ECG data using a bin averaging technique, ensuring consistency in data size between the two measurements.

### 2.4. Data Preprocessing and Dataset Construction

The measured data were preprocessed using Python 3.11.5. First, all three-axis ACC and ECG data were standardized using the StandardScaler function from the sklearn 1.4.1 module. The three-axis ACC data were preprocessed using the StandardScaler function to remove nan and empty values from the measured data, as the hardware used, the HiCardi+ instrument itself, had a denoising function. They were then aligned before the start time of each movement.

**Figure 6:**
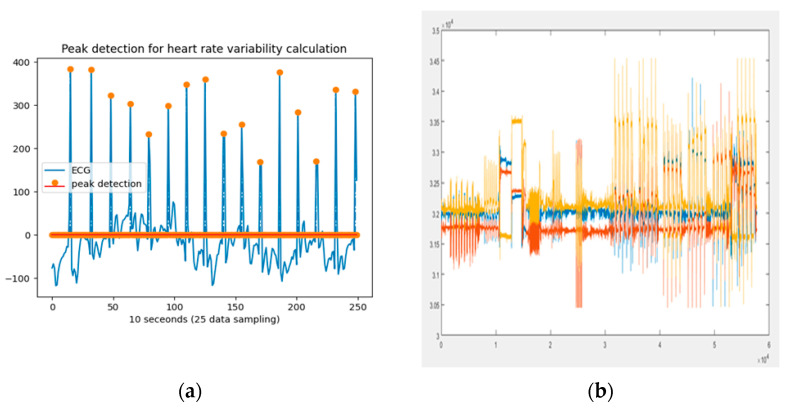
Example of measurement data image: (**a**) example of measurement ECG data and R peak detections and (**b**) example of measurement three−axis ACC data (red: *x*−axis; blue: *y*−axis; and yellow: *z*-axis).

The ECG data were first cleaned using the HiCardi+ device’s own denoising function, and then further denoising was performed by applying a 0.15 Hz high-pass filter and a 250 Hz low-pass filter to reduce signal fluctuations. HRV data were generated by calculating R peaks. The total duration of each movement was 10 s, including 5 s before and after the start, as plotted in Figure 6 with sampling data on the *x*-axis and mv on the *y*-axis.

HRV was calculated by identifying RR intervals within the segmented ECG data, converting them to milliseconds, and reconstructing them into one-dimensional data points.

This yielded 1050 data points per movement and a total of 13,650 datasets. The 15 movements were one-hot encoded using the to_categorical function in the keras 2.15.0 module to generate training and test datasets. A total of 1050 datasets were generated for each movement, for a total of 13,650 datasets. The training-to-test ratio was set to 0.7.

### 2.5. Implementation of a Behavioral Classification Model

**Figure 7:**
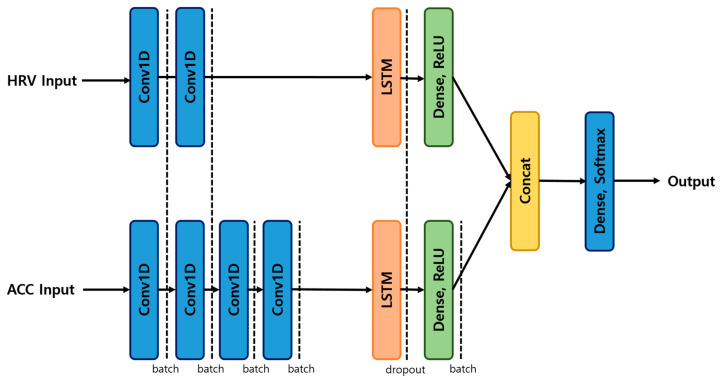
Model structure.

Figure 7 shows the procedure for classifying ACC and HRV data. In this study, we utilized data from 15 distinct movements, incorporating both ACC and HRV measurements. The ACC data were processed using a deep model, which combined a convolutional neural network (CNN) and a recurrent neural network (RNN) based on long short-term memory (LSTM). Simultaneously, the HRV data were processed using a wide model.

CNNs are widely used as one of the most suitable deep learning methods for the classification of time series data and image data. In this study, we leveraged the advantages of CNN while adding an LSTM layer to preserve the sequence of features over time. In particular, in the past few years, hybrid 1D-CNN-LSTM models have been widely used in the field of automatic classification of human emotions based on physiological signals, demonstrating that they can effectively analyze signals while preserving their temporal dependence [52,53,54,55,56].

For the ACC and HRV data, we developed a multi-input model based on the wide and deep architecture to compute precision, recall, and F1 scores for each dataset. The ACC data were processed using a convolutional neural network (Conv1D) in conjunction with a recurrent neural network (LSTM), with input dimensions of 250 × 3. The architecture included four Conv1D layers: the first layer employed 64 filters with a kernel size of 3, the second layer used 128 filters with a kernel size of 3, the third layer utilized 256 filters with a kernel size of 3, and the fourth layer incorporated 512 filters with a kernel size of 3. Following the LSTM layer, a dense layer with an ReLU activation function was applied.

For the HRV data, a similar architecture was used, consisting of a Conv1D and an LSTM network, with input dimensions of 26 × 2. The first Conv1D layer also used 64 filters and a kernel size of 3. The output from the LSTM layer was subsequently passed to a dense layer, which employed the ReLU activation function.

The features extracted from both the ACC data and the HRV input were concatenated into a single vector, which was subsequently fed into the final dense layer. The output of this layer was produced using the Softmax activation function. Based on this output, precision, recall, and F1 scores were calculated for each dataset to evaluate the performance of the model. The hyperparameters for the model in Figure 7 are shown in Table 2.

## 3. Results

**Figure 8:**
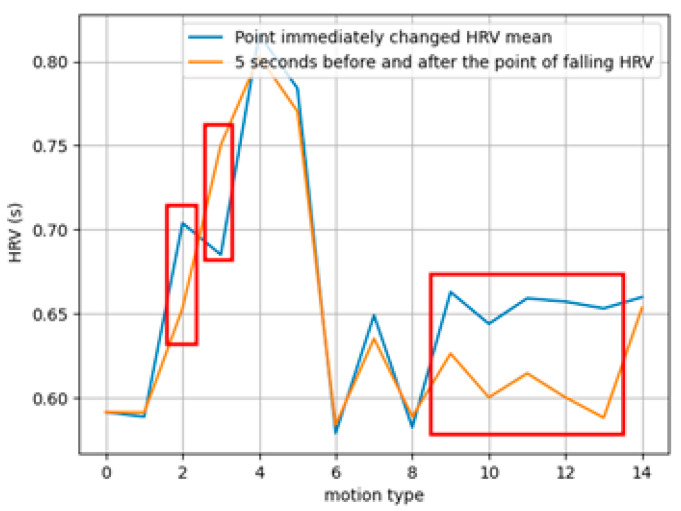
Comparison of heart rate variability according to variations in heart position due to movement. (The red boxes highlight regions where significant differences in HRV changes occur).

The physiological changes in HRV before and after the movements are shown in Figure 8. The most significant changes were observed in the standing, sitting, and falling postures, where differences in the potential energy of the heart were pronounced. This indicates that such changes are induced by baroreflex responses related to the movements, affecting the cardiovascular system [41].

By combining the ACC and HRV data into a single dataset, we evaluated the accuracy, recall, and F1 scores of the models and found that they performed significantly better than when the ACC and HRV data were used separately.

First, as shown in Table 3, the performance of the 15-motion classification models using only ACC data was rather poor, with an accuracy of 0.31, a recall of 0.33, and an F1 score of 0.31. On the other hand, the classification model based on HRV data achieved an accuracy of 0.82, a recall of 0.86, and an F1 score of 0.83, which was an improvement of +0.51, +0.53, and +0.52 over the ACC model, respectively.

**Figure 9:**
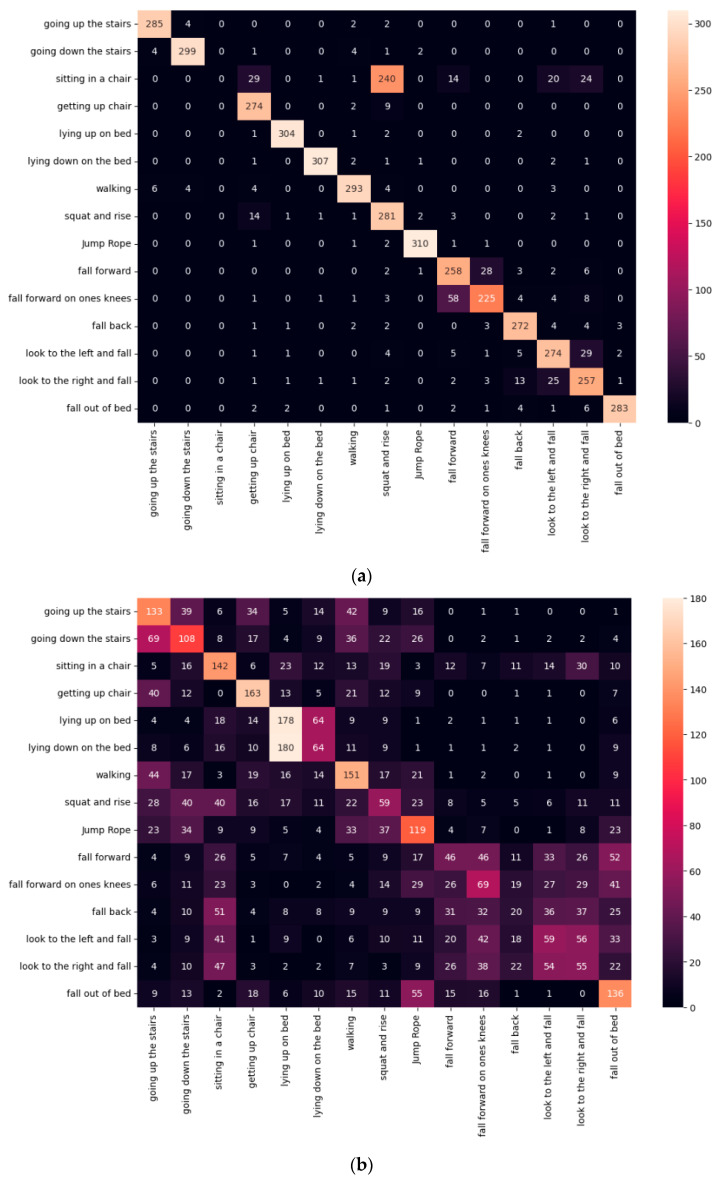

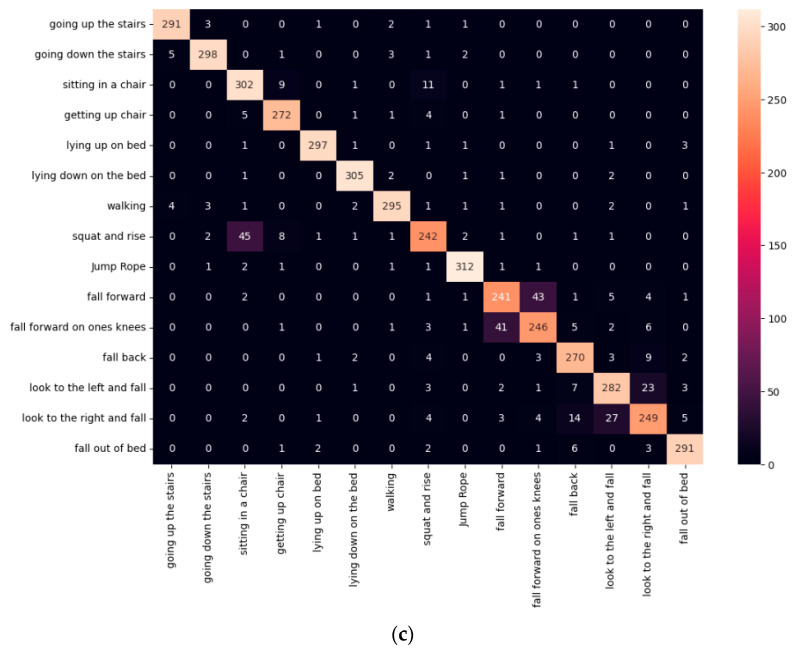
Heatmap results of the classification model by input data: (**a**) HRV data; (**b**) ACC data; and (**c**) ACC-HRV data.

In particular, the deep and wide model combining ACC and HRV data achieved a precision, recall, and F1 score of 0.91, which represented a performance improvement of over +0.60 over the ACC model. As shown in Figure 9, the model combining ACC and HRV data outperformed the HRV data alone by +0.09 in precision, +0.05 in recall, and +0.08 in F1 score.

These results strongly suggest that the performance of fall classification models can be significantly improved by integrating ACC data and HRV data. In particular, fall classification, which has been limited by ACC data alone, can provide a higher accuracy by combining HRV data.

## 4. Discussion

This study aimed to enhance the accuracy of fall classification by developing a deep and wide model that integrates multiple inputs, including HRV changes in conjunction with movement data. This approach facilitates the rapid classification and assessment of falls in daily life, a significant concern within the aging population. A total of 210 participants were recruited to measure data across 15 types of motion and train the model. The data were collected using HiCardi+, a Holter cardiograph from Mezoo’s, which simultaneously collected real-time electrocardiograms, acceleration data, and vital signs. It was attached to the subjects’ left side of the chest to measure data and had the advantage over other sensors of being able to provide more accurate data in close proximity to the heart. These high-precision data played an important role in improving the accuracy of the movement classification model in this study. Ultimately, the model achieved performance metrics of precision, recall, and F1 score of 0.91, reflecting a classification accuracy which was 0.60 higher than that of conventional models.

The physiological characteristics of the human body undergo changes with movement, and adjustments are made to maintain homeostasis in response to variations in both the internal and external environment [12]. In this context, positional changes in the heart induce pressure fluctuations within the cardiovascular system, with information being transmitted along the pressure vessels [41]. Consequently, HRV is dynamically influenced by changes in posture, such as sitting, lying down, and exercising [41,57,58,59]. While ACC data are strong in accurately reflecting the pattern and intensity of physical movement, they are limited in their ability to distinguish fine differences between similar movements. HRV data, on the other hand, reflect physiological signals that change with movement, providing additional information not available from ACC data. In particular, HRV can better distinguish subtle differences between movements through signals associated with changes in heart position [41,57,58,59].

Therefore, we believe that HRV can augment the physical movement data obtained from ACC data by providing complementary physiological features related to movements. This allowed us to use 13,650 datasets from 210 subjects, unlike previous studies, to overcome the limitations of single data, significantly improve the accuracy of analyses of 15 movement types, and more clearly distinguish details between similar movements [4,5,6,7,8,9,10,11,12,13,14,15,16,17,18,19,20,21,22,23,24,25,26,27,28,29,30,32,33,34,35,36,37,38]. Based on this, we propose that integrating HRV data, which vary with heart position, with ACC data can significantly improve classification accuracy, which could play a key role in important tasks such as fall classification.

The heatmap results of the model utilizing ACC data alone indicate difficulties in distinguishing between the movements ‘going up the stairs’ and ‘going down the stairs’, as well as between ‘lying down on the bed’ and ‘lying up on the bed’, and between ‘looking to the left and falling’ and ‘looking to the right and falling’. This is because these the movements have similar patterns of physical movement, and it is difficult to capture the details of the differences using ACC data alone. In contrast, the heatmap results from the deep and wide model that incorporates both ACC and HRV data demonstrate increased classification accuracy for the movements ‘going up the stairs’ and ‘going down the stairs’, ‘sitting in a chair’ and ‘getting up from a chair’, ‘walking’, ‘squatting and rising’, ‘jumping rope’, and ‘looking to the left and falling’ versus ‘looking to the right and falling’. Additionally, an improvement in accuracy for ‘lying down on the bed’ was also observed. These findings suggest that the incorporation of HRV data significantly enhances accuracy compared to using ACC data alone, indicating that HRV can effectively reflect changes in heart position and assist in movement classification.

This study has two primary limitations. The first limitation is that, although the deep and wide model utilizing both ACC and HRV data demonstrates a significant improvement in overall accuracy, it exhibits relatively low accuracy in distinguishing certain actions. Notably, the classification accuracy for ‘falling forward’ versus ‘falling forward on one knee’, as well as ‘looking left and falling’ versus ‘looking right and falling’, remains low. This may be attributed to the similarity between these actions, which poses challenges for the model in differentiating between them. To compensate for this limitation, future work will include data augmentation of four similar behaviors. The second limitation pertains to the participant selection, as the subjects included individuals beyond the elderly population. Given the differences in movement patterns between older adults and younger individuals, applying this study’s findings to individuals aged 65 and older may present challenges. However, the decision to include a broader age range was made to facilitate participant recruitment, as limiting the study to only elderly subjects could have hindered the availability of participants. This study was designed to classify a wide variety of movements to address this recruitment challenge.

In future research, we plan to build on the results of this study and improve the performance of the model using data augmentation techniques to improve the discrimination between similar movements.

In addition, we plan to conduct a follow-up study to validate the performance of the wide and deep model developed in this study by limiting the study to the elderly. By validating the model using elderly data to reflect the characteristics of the elderly, we will evaluate whether the model has reliability and generality across different age groups. In particular, we will check how effectively the model can handle physical activity limitations and vital sign irregularities that may occur in the elderly, and based on this, we will further optimize the model to expand its applicability.

This suggests that the performance of classification models can be enhanced by leveraging diverse motion and physiological information. Specifically, by considering the interplay between HRV and movement patterns, it becomes possible to predict falls more accurately across various indices.

## 5. Conclusions

The aim of this study was to enhance the accuracy of a fall classification model by utilizing real-time data to classify 15 distinct movements through a Holter electrocardiograph integrated with a three-axis acceleration sensor. To achieve this, the wide and deep model’s accuracy was improved by combining ACC data, which reflected the position of the heart, with HRV data that varied with movement. As a result, the accuracy of the model was found to be 0.60 and 0.09 higher compared to using ACC data and HRV data independently. Increased accuracy in classifying the 15 movements will allow for more precise detection and classification of falling movements in older adults. This can contribute to early detection of possible falls and appropriate preventive measures and enable a rapid response when a fall occurs. Therefore, improvements in movement classification accuracy are expected to have a positive impact on fall prevention.

## Figures and Tables

**Table 1:** Demographic characteristics of the participants.

**Age (Years, mean ± std)**	27.17 ± 4.48
**Gender**	210
**Male**	115
**Female**	95
**Height (cm, mean ± std)**	169.97 ± 8.23
**Weight (kg, mean ± std)**	65.84 ± 13.0

**Table 2:** Model’s hyperparameters.

Hyperparameters	Value
Conv1D filter size	64, 128, 256, 512
Conv1D kernel size	3
LSTM units (ACC data)	512, 256, 126
LSTM units (HRV data)	64
Dropout rate	0.3 (LSTM), 0.5 (Dense)
Dense layer units	64
Activation functions	ReLU, Softmax
L2 regularization	0.01
Output class size	15
Learning rate	0.001
ReduceLROnPlateau patience	10
EarlyStopping patience	20

**Table 3:** Classification accuracy model results by data.

	HRV Data	ACC Data	ACC-HRV Data
**Precision**	0.82	0.31	0.91
**Recall**	0.86	0.33	0.91
**F1 score**	0.83	0.31	0.91

## Data Availability

The data presented in this study are provided at the request of the corresponding author due to privacy and confidentiality concerns.
